# All-optical tunable buffering with coupled ultra-high *Q* whispering gallery mode microcavities

**DOI:** 10.1038/s41598-017-10035-4

**Published:** 2017-09-06

**Authors:** Wataru Yoshiki, Yoshihiro Honda, Tomohiro Tetsumoto, Kentaro Furusawa, Norihiko Sekine, Takasumi Tanabe

**Affiliations:** 1 Department of Electronics and Electrical Engineering, Faculty of Science and Technology, Keio University, 3-14-1, Hiyoshi, Kohoku-ku, Yokohama 223-8522 Japan; 2 Advanced ICT Research Institute, National Institute of Information and Communications Technology, 4-2-1 Nukuikitamachi, Koganei City, Tokyo 184-8795 Japan

## Abstract

All-optical tunable buffering was recently achieved on a chip by using dynamically tuned coupled mode induced transparency, which is an optical analogue of electromagnetically induced transparency. However, the small *Q* s of about 10^5^ used in those systems were limiting the maximum buffering time to a few hundred ps. Although employing an ultra-high *Q* whispering gallery mode (WGM) microcavity can significantly improve the maximum buffering time, the dynamic tuning of the WGM has remained challenging because thermo-optic and pressure tunings, which are widely used for WGM microcavities, have a very slow response. Here we demonstrate all-optical tunable buffering utilizing coupled ultra-high *Q* WGM cavities and the Kerr effect. The Kerr effect can change the refractive index instantaneously, and this allowed us to tune the WGM cavity very quickly. In addition, from among the various WGM cavities we employed a silica toroid microcavity for our experiments because it has an ultra-high *Q* factor (>2 × 10^7^) and a small mode volume, and can be fabricated on a chip. Use of the Kerr effect and the silica toroid microcavity enabled us to observe an on-chip all-optical tunable buffering operation and achieve a maximum buffering time of 20 ns.

## Introduction

The ability to capture light in a tiny space and buffer it for a certain time period is crucial for various applications including all-optical signal processing^1^. A major and powerful platform for all-optical buffering is electromagnetically induced transparency (EIT)^2^ in which a narrow transmission window is formed in its absorption spectrum under irradiation from a control laser. In an EIT system, signal pulse information is coherently transferred into atoms and stored for a very long time (for instance, >1 min^3^). Recently, it was proven that coupled optical modes in optical cavities are capable of exhibiting an EIT-like phenomenon, which is called CMIT^4^. In contrast to EIT, CMIT can be achieved at room temperature and does not require any atom-trapping techniques thus paving the way for the practical use of CMIT for all-optical buffering. Moreover, if CMIT is developed with micro- or nano-cavities, we can realize an all-optical buffer on a chip with a very small footprint^5, 6^. These features differentiate a CMIT-based all-optical buffer from fibre-based buffers^7, 8^, which often require a fibre length of a few hundred meters. More recently, even all-optical “tunable” buffering has become possible with CMIT. It was revealed theoretically that the dynamic tuning of the cavity resonance provides CMIT-based all-optical buffering with tunability^9^. Later, this theoretical prediction was confirmed experimentally with coupled silicon microring cavities^10^. However, the maximum buffering time is limited to a few hundred ps in this study because the *Q* factor of the cavity remains very low (~10^5^).

A simple way to achieve a long buffering time is to employ ultra-high *Q* WGM microcavities. WGM is formed when the light wave propagates along the side-wall of a circular cavity by total internal reflection. WGM microcavities are usually fabricated with highly transparent materials (e.g. silica, CaF_2_, and MgF_2_) and have a smooth surface, and so they exhibit a *Q* of over 10^8^ ^11^. CMIT has already been reported in various WGM cavities^12–15^ and employed for various applications including an isolator^16, 17^, sensing^18^, optomechanics^19^, and microwave photonics^20^. Nonetheless, using it for all-optical tunable buffering is still challenging because it is not easy to tune the resonance of WGM cavities “dynamically”. This is because, for one thing, WGM cavities are not compatible with carrier injection and excitation, which are major methods for tuning the cavity resonance very fast. In addition, although thermo-optic and pressure-induced tuning techniques are available for WGM microcavities^21–23^, they are too slow to be employed for all-optical tunable buffering. If we can tune the resonance of an ultra-high *Q* WGM microcavity very quickly, it is possible to achieve all-optical tunable buffering with a long buffering time.

In this letter, we describe an experimental demonstration of all-optical tunable buffering with coupled ultra-high *Q* microcavities. To tune the resonance we employed the Kerr effect, which can change the refractive index almost instantaneously. This enabled us to tune the resonance of the WGM cavity very fast and achieve all-optical tunable buffering. Although the Kerr effect has already been observed in WGM cavities and applied to all-optical switching and frequency conversion^24–27^, this is first time it has been employed to tune CMIT. From among various WGM cavities we chose a silica toroid microcavity as the platform for our experiments because it has an ultra-high *Q* factor (>2 × 10^7^) and a small mode volume, and can be fabricated on a chip^28^. Taking advantage of the silica toroid microcavity and the Kerr effect, we achieved a maximum buffering time of 20 ns.

## Results

### Concept and numerical analysis

**Figure 1:**
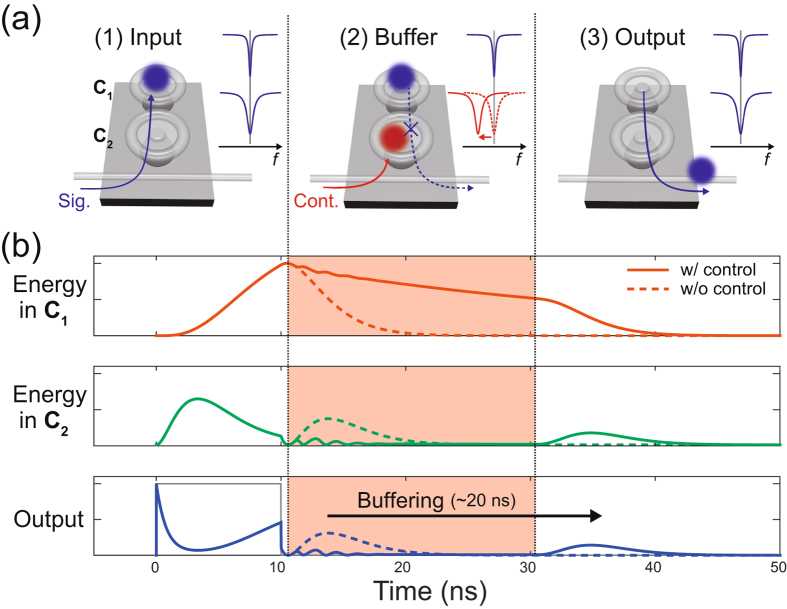
Basic concept of all-optical tunable buffering with coupled silica toroid microcavities. (**a**) Schematic illustration of the buffering operation. The system consists of two cavities denoted C_1_ and C_2_, and only C_2_ couples to the tapered optical fibre. (**b**) Numerical results of the buffering operation. The top, middle, and bottom graphs represent the signal light energies in C_1_ and C_2_, and the output signal power, respectively. The solid and dashed lines are calculated with and without control lights, respectively. The control light is being input in the red region, and the solid grey line in the bottom graph represents the input signal power. The intrinsic *Q*s of C_1_ and C_2_, and the coupling rate between C_1_ and C_2_ are set at 5 × 10^7^, 2 × 10^6^, and 2*π* × 96 MHz, respectively. The peak power of the control pulse is 10 W.

First of all, we describe the basic concept behind all-optical tunable buffering with coupled silica toroid microcavities. A situation is considered in which two silica toroid microcavities couple together as shown in Fig. 1(a). If the resonant frequencies of the two cavities (C_1_ and C_2_) are the same, the light input into a tapered optical fibre (i.e. signal light) first couples to C_2_, and then reaches C_1_ (see “(1) input” in the figure). While the light remains in C_1_, an additional high-power light (i.e. control light) is input into another mode in C_2_ as illustrated in “(2) buffer” in the figure. The input control light induces the Kerr effect, and then tunes the resonance of C_2_, which creates a mismatch between the resonances of C_1_ and C_2_. This mismatch prevents the signal light in C_1_ from escaping to C_2_, and so light is captured in C_1_ while the control light is being input. After the control light is turned off, the resonances of the two cavities become matched again. In this condition, the signal light in C_1_ is allowed to couple into C_2_, and it is then output to a tapered optical fibre (“(3) output”). Therefore, the signal light can be buffered for an arbitrary time period by changing the duration of the control pulse. This is the concept of all-optical tunable buffering with coupled silica toroid microcavities.

We performed a numerical calculation to confirm the validity of this concept. The results are shown in Fig. 1(b). When there is no control light, the signal light first couples to C_2_, and then moves to C_1_ (the dashed lines in the graphs). As soon as the light energy in C_1_ reaches its maximum value, it starts to return to C_2_. Eventually, the light in C_2_ is output to the fibre and creates a second peak in the output. On the other hand, if the control light is input (shown as the red regions), the light in C_1_ does not couple back to C_2_ and is stored in C_1_ due the mismatch of the resonant frequencies. After the control light is turned off, the light again begins to transfer from C_1_ to C_2_, and then creates the second peak in the output. Compared with the case with no control light, the second peak is delayed for 20 ns, which agrees well with the time width of the control input. This means that the position of the second peak can be arbitrary controlled with the control input, and the concept of all-optical tunable buffering is feasible.

Then, we discuss how to maximize the performance of the all-optical tunable buffering. The performance is basically evaluated using two factors, namely the maximum buffering time and the bandwidth. To increase the maximum buffering time, we should make the *Q* factor of C_1_ as high as possible. As seen in the top graph in Fig. 1(b), the light energy in C_1_ is decaying while the control light is being input. This decay rate corresponds to the intrinsic cavity loss rate of C_1_, which is inversely proportional to the *Q* value of C_1_. If the signal light dissipates completely while the control light is being input, no signal light can be extracted from the output even after the control light is turned off. Thus, a *Q* of C_1_ limits the maximum buffering time and should be set as high as possible. For instance, when we target a holding time of 0.1 *μ*s, we need a *Q* of 1.2 × 10^8^. In addition, the buffer should have a broad bandwidth; in other words, it should be able to respond to a signal light with a short pulse width. It is intuitive to assume that to capture a short signal light we should minimize the time taken by the signal light to reach C_1_. This time can be divided into two parts, namely the time taken to couple from the fibre to C_2_ and that taken to move from C_2_ to C_1_. Generally, the former and latter times are determined by the *Q* of C_2_ and the coupling rate between C_1_ and C_2_, respectively. Therefore, lowering the *Q* of C_2_ and increasing the coupling rate lead to a broader bandwidth. However, it should be noted that a larger frequency shift is needed to make the resonances of C_1_ and C_2_ mismatched in the case of a lower *Q* value for C_2_ and a higher coupling rate between C_1_ and C_2_. It is intuitive to assume that we have to input a higher control power into the cavity to induce a larger frequency shift. Thus, there is a trade-off between the bandwidth and the control power, and in practice the trade-off sets the upper limit of the bandwidth. The parameters of the coupled silica toroid microcavities must be set after considering the above discussion.

### Sample preparation and characterization

**Figure 2:**
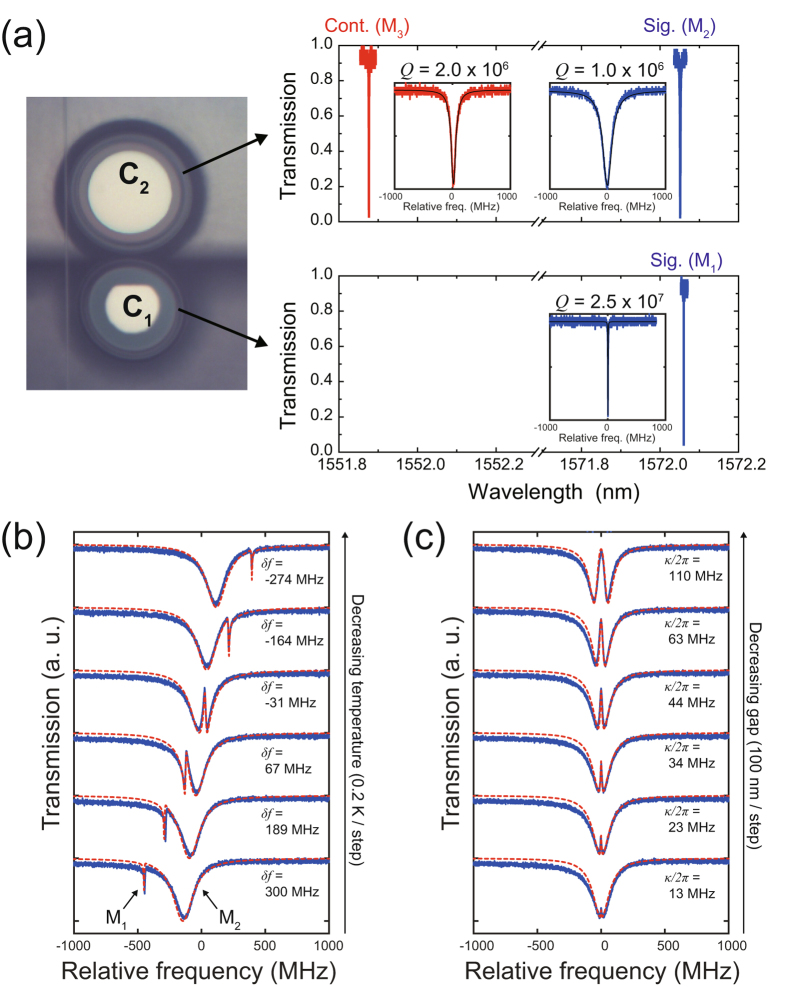
Preparing coupled silica toroid microcavities. (**a**) Characterization of fabricated samples. The left picture is a microscope image of C_1_ and C_2_. The top and bottom graphs represent the transmission spectra of optical modes in C_2_ and C_1_, respectively. The blue and red lines are the transmission spectra of the signal (M_1_ and M_2_) and control (M_3_) modes. The insets in the graphs are enlargements of the transmission spectra. (**b**,**c**) The transmission spectra of coupled M_1_ and M_2_ for different (**b**) temperatures of C_1_ and (**c**) different gaps between C_1_ and C_2_, respectively. The blue solid and red dashed lines represent the measured and fitted data, respectively. The frequency mismatch between M_1_ and M_2_ (*δf*) and the coupling rate between M_1_ and M_2_ (*κ*/2*π*) were estimated from the fitted data.

To couple two silica toroid microcavities, we fabricated them near the edges of separate Si chips^29^. This enabled us to place two microcavities very close together (<1 *μ*m) as shown in Fig. 2(a) because the toroidal part of the cavity protrudes from the chip. The transmission spectra of the modes that were used for the experiments are shown in the graphs in Fig. 2(a). We selected two modes (M_1_ and M_2_) for the signal light and a mode (M_3_) for the control light from the optical modes in C_1_ and C_2_. M_1_ and M_2_ couple together, and so the signal light input into M_2_ is transferred into M_1_ as illustrated in Fig. 1. On the other hand, M_3_ does not couple to any modes because it is used only for inducing the Kerr effect in C_2_. As discussed in the previous subsection, the *Q* factor of M_1_ should be higher to obtain a longer maximum buffering time because the signal light is stored in M_1_ during the buffering operation. Taking this into consideration, we chose a mode with a *Q* of over 2 × 10^7^ for M_1_. On the other hand, the *Q* factor of M_2_ and M_3_ should have a moderate value. This is because a lower *Q* results not only in a larger bandwidth but also in a higher required control power. In addition, if M_3_ is chosen from the same mode family as M_2_, it should have a comparable *Q* factor to that of M_2_. Hence, we employed modes with a *Q* of around 10^6^ for M_2_ and M_3_.

Next, we tuned the resonant frequency of M_1_ to that of M_2_ by changing the temperature of C_1_, and then achieved coupling between the two modes. Figure 2(b) shows the transmission spectra of the coupled M_1_ and M_2_ for a different temperature. As seen from each graph in the figure, there is a split in the transmission spectrum, which is direct evidence of coupling between the two modes. In addition, it shows a clear Fano resonance, which usually occurs when optical modes with very different *Q* factors are coupled^30^. The transmission spectra for the different gaps between the two cavities are shown in Fig. 2(c). The split width (the coupling rate between M_1_ and M_2_) becomes larger as the gap decreases, which means that the coupling rate is arbitrarily adjustable with the gap. In the buffering experiments, we completely matched the resonance frequencies of M_1_ and M_2_ and adjusted the split width to around 70 MHz. This is because too large a split width (i.e. a higher coupling rate) results in the need for higher control power as discussed in the previous subsection.

### Demonstration of all-optical tunable buffering

**Figure 3:**
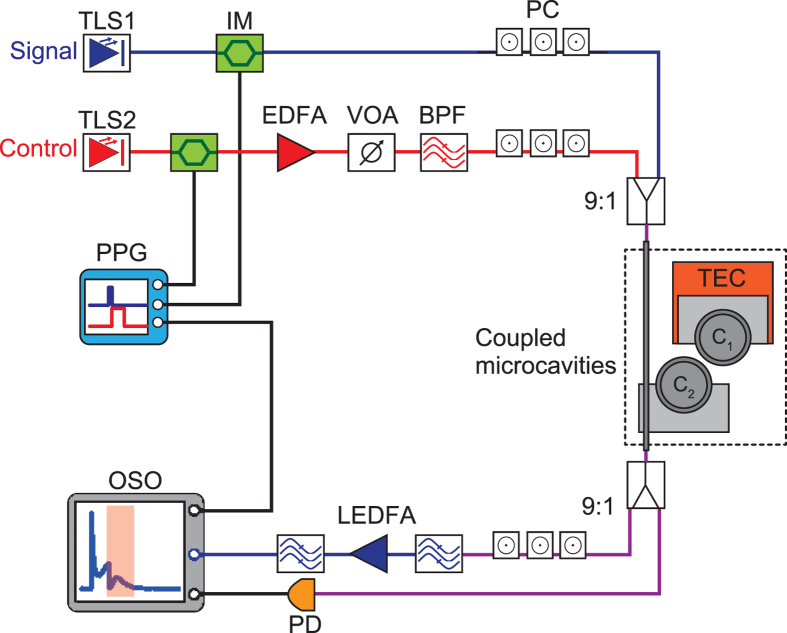
Setup for all-optical tunable buffering. TLS: Tunable laser source. IM: Intensity modulator. EDFA: Erbium-doped fibre amplifier. VOA: Variable optical attenuator. BPF: Band pass filter. PC: Polarization controller. TEC: Thermoelectric cooler. LEDFA: L-band EDFA. PD: Photodetector. OSO: Optical sampling oscilloscope. PPG: Pulse pattern generator.

Here we describe the experimental results we obtained for all-optical tunable buffering. Figure 3 shows our experimental configuration. We employed two tunable laser sources (TLSs) for the emitting signal and control lights. Both lights were modulated into rectangular pulses with intensity modulators (IMs). After modulation, the control light was amplified with an erbium-doped fibre amplifier (EDFA). Both lights were input into the coupled silica toroid microcavities via a tapered optical fibre^31^, and at the output the signal light alone was detected with an optical sampling oscilloscope (OSO) after amplification with an L-band EDFA (LEDFA). Note that the output control light was filtered out with a band pass filter (BPF) before the amplification.

**Figure 4:**
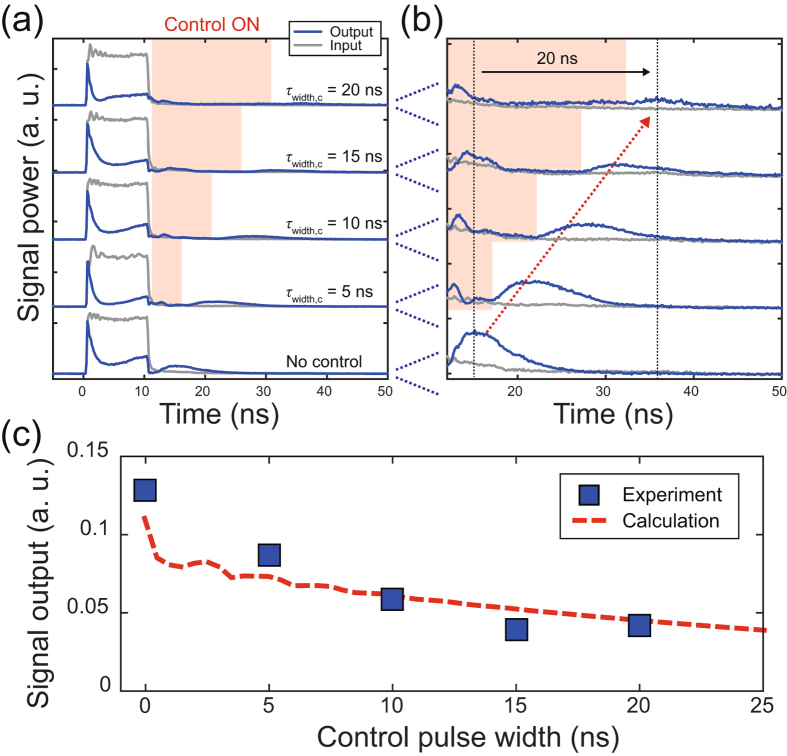
Experimental results of all-optical tunable buffering. (**a**) Signal output for different control pulse widths *τ*_width,c_. The blue and grey solid lines represent the signal output and input, respectively. The time width of the input signal pulse is 10 ns. The pulse widths and the peak powers of the control pulses (*τ*_width,c_, *P*_in,c_) are (5 ns, 15.1 W), (10 ns, 13 W), (15 ns, 8.4 W), and (20 ns, 7.3 W), respectively. The control pulse is input in the red region. (**b**) Enlargement of (**a**). (**c**) The peak power of the buffered signal pulse as a function of the control pulse width. The blue rectangles and red dashed lines plot the measured and calculated results, respectively. The same parameters used in Fig. 2(b,c) were employed for the calculation. *y* axis is normalized by the peak power of the input signal pulse.

The experimental results are presented in Fig. 4. We measured the signal output for the control light with different pulse widths as shown in Fig. 4(a,b). As seen from the figures, all-optical tunable buffering appears to work well. The signal light pulse is stored while the control light is being input (indicated by the red region), and then it is coupled out once the control light has been turned off. In addition, the figures also indicate that the buffering times can be controlled by changing the control pulse widths. We can buffer the signal pulse with a time width of 10 ns for up to 20 ns in the experiment. These results are direct evidence proving that all-optical tunable buffering was achieved. Close observation of Fig. 4(b) implies that a small amount of the signal light leaks out while the control light is being turned on. This is owing to the small coupling that remains between M_1_ and M_2_, which allows the signal light to escape to the tapered optical fibre through M_2_. However, the signal light leakage is believed to be small enough to have no influence on the buffering performance. This is because there was no change in the leakage when the input control power (i.e. the amount of resonance shift) was changed. In other words, the dominant loss source during the buffering must be the intrinsic loss of M_1_.

The blue rectangles in Fig. 4(c) plot the peak power of the signal output for different control pulse widths. The signal output becomes smaller as the control pulse width increases, and it is around 8 times smaller (output efficiency of 13%) than the peak power of the input signal pulse even when the control pulse width is 0 ns. This is because the spectrum of the input signal pulse never completely matches the transmission spectrum of the coupled microcavities. This issue is common to applications utilizing a resonant structure. The figure shows that the power becomes about 3 times smaller with a control pulse width of 20 ns than with a width of 0 ns. This attenuation corresponds to a propagation loss of 1.14 dB/m. Considering the fact that even the latest on-chip “fixed” buffers exhibit a propagation loss of about 0.1 dB/m^32^, this value appears to be reasonable. It should be emphasized that the red dashed curve in the figure plots the calculated results and agrees well with the experimental results.

## Discussion

Here we compare the results we obtained with those of previous studies. A CMIT-based all-optical tunable buffer is advantageous because of its small size and capacity for on-chip fabrication. However, the previously reported buffer^10^ is composed of low-*Q* (~10^5^) cavities, thus its maximum buffering time is limited to a few hundred ps. Although there are other kinds of on-chip all-optical tunable buffering, which are achieved with the dynamic tuning of a waveguide-coupled photonic crystal nanocavity^33, 34^ and a photonic crystal slow light waveguide^35^, they also have an issue in terms of buffering time. Conversely, the all-optical tunable buffering used in this study exhibited a buffering time of 20 ns thanks to the ultra-high *Q* factor of the silica toroid microcavity. Therefore, we can conclude that our buffer is advantageous as regards buffering time.

On the other hand, our buffer has a narrower signal light bandwidth than previous studies. We buffered a 10-ns signal pulse while the buffering of an optical pulse with a width of less than 100 ps has been reported in previous studies^10, 33–35^. As discussed previously, although we can increase the bandwidth by decreasing the *Q* value of C_2_ and strengthening the coupling, this approach results in a higher required control power. Thus we need a way to solve the trade-off between bandwidth and control power. There are several ways of accomplishing this. The first is to coat the silica toroid microcavity with a highly nonlinear material such as plasmonic particles, graphene, or carbon nanotubes (CNTs)^36–38^. The size of the Kerr effect is in proportion to the nonlinear refractive index *n*_2_, and so we can increase the frequency shift by enhancing the *n*_2_ value even if the input control power is fixed. Actually, the enhancement of four-wave mixing was observed experimentally in a CNT/polymer composite-coated tapered optical fibre^38^. Another way is to combine microcavities fabricated with different materials. For example, if a silicon microcavity and a silica toroid microcavity are employed for C_2_ and C_1_, respectively, we can utilize the carrier-plasma effect to tune the resonance of C_2_ quickly and broadly while maintaining the ultra-high *Q* of C_1_. In fact, the integration of a silicon platform and an ultra-high *Q* WGM cavity was recently reported^39^. We believe that we can further improve the bandwidth by taking advantage of these methods.

It should be noted that, in addition to the previous studies reviewed so far, all-optical buffering with optomechanically induced transparency (OMIT) was recently reported^40–42^. In contrast to CMIT in which two optical modes couple, OMIT is achieved by coupling between optical and mechanical modes in the microcavity. A mechanical mode generally has a much longer lifetime than an optical mode. Hence these devices can buffer light for a long time (>10 *μ*s) thanks to the long lifetime of the mechanical mode. However, the coupling between the optical and mechanical modes is not usually very strong, which leads to a very narrow bandwidth (<1 MHz). In addition, they are achieved with a silica microsphere, which is difficult to fabricate on a chip. Therefore, our buffer is advantageous in terms of bandwidth and its capacity for on-chip fabrication.

## Summary

In summary, we have presented an experimental demonstration of all-optical tunable buffering with coupled ultra-high *Q* microcavities. The use of the Kerr effect, which can shift the resonance of the cavity almost instantaneously, made it possible to achieve all-optical tunable buffering with coupled ultra-high *Q* WGM microcavities^24–27^. It should be noted that this is first time that the Kerr effect has been employed for tuning coupled microcavities. We chose a silica toroid microcavity as the platform for our experiments because it has an ultra-high *Q* factor (>2 × 10^7^) and a small mode volume, and can be fabricated on a chip^28^. Thanks to the ultra-high *Q* factor and the Kerr effect, the maximum buffering time reached 20 ns, which was limited to a few hundred ps in previous studies^10, 33–35^. Although the observed bandwidth was narrower than those of previous studies, we believe that it can be improved by coating the silica toroid microcavity with material with a high *n*_2_ value or achieving CMIT using microcavities fabricated with different materials.

## Methods

### Numerical model

The numerical model is based on coupled mode theory^15, 43^. According to the theory, the amplitudes of M_1_ and M_2_ (*a*_1_, *a*_2_) are given as

1 $$\frac{{\rm{d}}{a}_{1}(t)}{{\rm{d}}t}=[j{\omega }_{1}-\frac{{\gamma }_{{\rm{int1}}}}{2}]\,{a}_{1}(t)+j\frac{\kappa }{2}{a}_{2}(t),$$

2 $$\frac{{\rm{d}}{a}_{2}(t)}{{\rm{d}}t}=[j({\omega }_{2}+\delta \omega (t))-\frac{{\gamma }_{{\rm{int2}}}+{\gamma }_{{\rm{wav2}}}}{2}]\,{a}_{2}(t)+j\frac{\kappa }{2}{a}_{1}(t)+\sqrt{{\gamma }_{{\rm{wav2}}}}{s}_{{\rm{in}}}(t),$$

where *ω*_*i*_, *γ*_int*i*_, and *γ*_wav*i*_ are the resonant angular frequency, the intrinsic loss rate, and the waveguide coupling rate of M_*i*=1,2_, respectively. *s*_in_ is the amplitude of the signal input and appears only in Eq. (2) because the tapered optical fibre is coupled only to C_2_. *κ* is the coupling rate between M_1_ and M_2_, and *κ*/2*π* corresponds to the split in the transmission spectrum. *δω*(*t*) is the shift of the resonant angular frequency induced by the control light via the Kerr effect^27^. The amplitude of the output *s*_out_ is given as ${s}_{{\rm{out}}}={s}_{{\rm{in}}}-\sqrt{{\gamma }_{{\rm{wav2}}}}{a}_{2}$. Note that the light energy in M_1_ and M_2_, and the output signal power are equal to |*a*_1_|^2^, |*a*_2_|^2^, and |*s*_out_|^2^, respectively.

### Fabrication

The silica toroid microcavities employed in the experiments (C_1_ and C _2_) were fabricated using five processes: (1) photolithography, (2) HF etching, (3) dicing, (4) XeF_2_ dry etching, and (5) laser reflow^28^. With the photolithography process (1), circular resist patterns 100 *μ*m in diameter were developed on a silicon chip with a 2 *μ*m-thick silica layer. Then, (2) HF etching removed only the parts of the silica layer that were not covered with the resist patterns. As a result, circular silica pads were formed on the silicon chip. Next, by using a high-precision dicing machine (DISCO DAD3430) we cut part of the chip to position the silica pads near the edge of the chip ((3) dicing process). After that, (4) XeF_2_ dry etching was used to undercut the silica pads, and then silica disks on a silicon pillar were fabricated. Because the pads were placed near the edge, parts of the disks protruded from the chip as required for coupling two microcavities. Finally, the fabrication was completed by irradiating the silica disks with a CO_2_ laser and annealing them so that their surface became very smooth ((5) laser reflow process). Note that C_1_ and C_2_ were fabricated on different chips to control the gap between them during the characterization.

### Coupling silica toroid microcavities

We first measured the transmission spectra of C_1_ and C_2_ individually. We coupled light emitted by a TLS (Santec TSL-710, linewidth of 100 kHz) into the cavity via the tapered optical fibre and detected the output light with a PD (Thorlabs DET08CFC, bandwidth of 5 GHz). By scanning the laser frequency, we obtained the transmission spectra. Note that we employed a Mach-Zehnder interferometer (NTT Electronics, free spectral range of 1 GHz) as a frequency reference. From the obtained transmission spectra of C_1_ and C_2_, we selected three optical modes for M_1_, M_2_, and M_3_. The resonant wavelengths of M_1_ and M_2_ should be close (e.g. 100 pm) to make M_1_ easy to tune to M_2_. Then, we placed the two chips on which C_1_ and C_2_ were fabricated on automatic *xyz* stages (resolution of 10 nm) separately to precisely control the gap between the two chips (the two cavities). To tune M_1_ to M_2_ we changed the temperature of C_1_ with a TEC whose temperature was stabilized with a TEC driver (Thorlabs TED200C, temperature stability of 2 mK). We covered the setup with a hood to make the temperature environment stable and achieve good repeatability of the measurement.

### Data availability

The datasets generated during the current study are available on request from the corresponding author.
